# MiRNA-320 in the human follicular fluid is associated with embryo quality in vivo and affects mouse embryonic development in vitro

**DOI:** 10.1038/srep08689

**Published:** 2015-03-03

**Authors:** Ruizhi Feng, Qing Sang, Yan Zhu, Wei Fu, Miao Liu, Yan Xu, Huijuan Shi, Yao Xu, Ronggui Qu, Renjie Chai, Ruijin Shao, Li Jin, Lin He, Xiaoxi Sun, Lei Wang

**Affiliations:** 1 State Key Laboratory of Genetic Engineering and MOE Key Laboratory of Contemporary Anthropology, School of Life Sciences, Fudan University, Shanghai, China; 2 Institutes of Biomedical Sciences, Fudan University, Shanghai, China; 3 Shanghai Ji Ai Genetics and IVF Institute, Obstetrics and Gynecology Hospital, Fudan University, Shanghai, China; 4 Guangdong No.2 provincial people's hospital, Guangzhou, China; 5 Shanghai Institute of Planned Parenthood Research, Shanghai, China; 6 Key Laboratory for Developmental Genes and Human Disease, Ministry of Education, Institute of Life Sciences, Southeast University, Nanjing 210096, China; 7 Bio-X Center, Key Laboratory for the Genetics of Developmental and Neuropsychiatric Disorders, Ministry of Education, Shanghai Jiao Tong University, Shanghai, China; 8 Department of Physiology/Endocrinology, Institute of Neuroscience and Physiology, University of Gothenburg, Gothenburg, Sweden

## Abstract

Previous work from our laboratory demonstrated the existence of miRNAs in human follicular fluid. In the current study, we have sought to identify miRNAs that might affect oocyte/embryo quality in patients undergoing intracytoplasmic sperm injection and to investigate their roles in *in vitro* fertilization outcomes in mouse oocytes. 53 samples were classified as Group 1 (high quality) if the day-3 embryos had seven and more cells or as Group 2 (low quality) if the embryos had six and fewer cells. TaqMan Human microRNAs cards and qRT-PCR were performed to verify differently expressed miRNAs. The function of the corresponding miRNA was investigated in mouse oocytes by injecting them with miRNA-inhibitor oligonucleotides. We found that hsa-miR-320a and hsa-miR-197 had significantly higher expression levels in the Group 1 follicular fluids than in Group 2 (*p* = 0.0073 and *p* = 0.008, respectively). Knockdown of mmu-miR-320 in mouse oocytes strongly decreased the proportions of MII oocytes that developed into two-cell and blastocyst stage embryos (*p* = 0.0048 and *p* = 0.0069, respectively). Wnt signaling pathway components had abnormal expression level in miR-320 inhibitor-injected oocytes. This study provides the first evidence that miRNAs in human follicular fluid are indicative of and can influence embryo quality.

The follicular fluid provides the microenvironment in which oocytes develop, mature, and ovulate^1^. It contains various kinds of hormones, proteins, metabolites, and regulatory molecules that play critical roles in the development and maturation of oocytes^2^. With the advent of assisted reproduction techniques (ART), follicular fluid has become a daily by-product of controlled ovarian hyperstimulation and oocyte retrieval. Although the number of in vitro fertilization (IVF) procedures is increasing every year, pregnancy rates using ART are still far less than desirable. It has been estimated that only about 30% of IVF cycles result in a pregnancy^3^. To improve ART outcome, one fundamental problem that needs to be solved is to accurately predict oocyte and embryo developmental potential. Because the follicular fluid affects oocyte development, its composition has been investigated as a possible predictor of oocyte and embryo quality. Previous studies have shown the relationships between growth factors^4^,^5^,^6^, proteins^7^,^8^, reactive oxygen species^9^,^10^, and metabolites^11^,^12^ in the follicular fluid and oocyte quality, fertilization rate, embryonic developmental potential, and pregnancy outcome^13^.

MiRNAs, which are typically about 22 nucleotides long, act as small post-transcriptional regulatory molecules that function by binding to their specific mRNA targets, directly degenerating mRNAs or inhibiting their translation to proteins^14^. MiRNAs play important roles in many physiological processes and have been implicated in numerous diseases^15^. Although miRNAs have been extensively investigated in other body fluids such as serum and plasma^16^,^17^, research on miRNAs in the follicular fluid is in its infancy.

In our previous work, we demonstrated for the first time the existence of miRNAs in human follicular fluid and determined their in vitro roles in steroidogenesis and their in vivo roles in polycystic ovary syndrome (PCOS)^18^. Recently, Santonocito et al. and Diez-Fraile et al. also independently reported microRNAs existing in human follicular fluids^19^,^20^. However, until now there have been no reports on the role of follicular fluid miRNAs in the developmental potential of oocytes and embryos.

In the present study, we collected follicular fluid from the very first single aspirated follicle of intracytoplasmic sperm injection (ICSI) patients. Oocyte development, fertilization outcome, and embryo quality at three days after insemination were recorded and evaluated. We produced differentiated miRNA expression profiles of the follicular fluid and used qRT-PCR to identify miRNAs associated with embryonic development. Finally, we investigated the effects and molecular mechanisms of corresponding miRNAs in oocyte fertilization and embryonic development by injecting inhibitor oligonucleotides into mouse metaphase-II (MII) oocytes.

## Results

### Oocyte/embryo development outcomes and clinical characteristics

**Figure 1:**
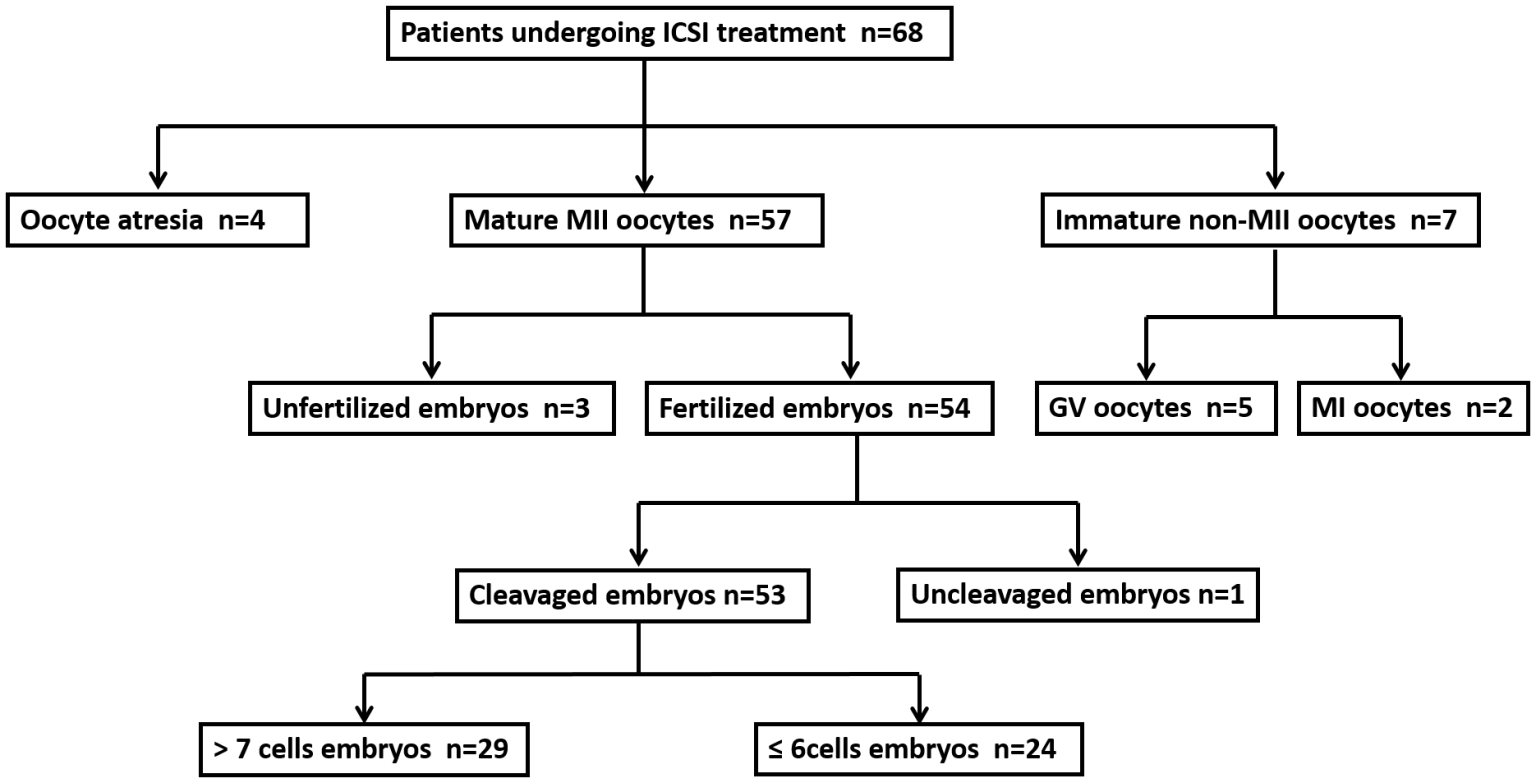
Flow chart for selecting samples, collecting samples, and choosing those to be included in the following experiments according to their status and destiny. ICSI, intracytoplasmic sperm injection. GV, germinal vesicle. MI, metaphase I. MII, metaphase II.

A flow diagram giving an overview of the samples is provided in Figure 1. Sixty-eight follicular fluid samples and matched oocytes were collected from 68 women with tubal factor or male factor infertility. Of the 68 oocytes, there were seven immature oocytes (GV stage or MI stage) and four atresic oocytes, and these were excluded from the following ICSI treatment. Of the 57 mature oocytes undergoing ICSI treatment, 53 formed two pronuclei 14–16 hours after the treatment. These 53 follicular fluid samples and matched mature MII oocytes with normal fertilization outcome were used to identify miRNAs associated with oocyte/embryo development potential. The basic clinical characteristics, hormone levels, and oocyte and embryo retrieval outcomes of these patients are summarized in Table 1. There were no significant differences for any of the clinical characteristics or hormone levels between the two groups. In addition, no significant differences were found regarding the number of oocyte/embryo retrieval results between the two groups.

### MiRNA profiling and the identification of differentially expressed miRNAs associated with embryo quality

We pooled 200 μL of each sample of follicular fluid in Group 1 and Group 2 and extracted the corresponding total RNA from the pooled samples. High expression-level miRNAs (Raw Ct < 30) are listed in Supplementary Table 1. In general, miRNA expression level in Group 1 was greater than in Group 2. Because embryo quality decreased from Group 1 to Group 2, miRNAs whose expression levels change between Group 1 and Group 2 might play a role in embryo development potential. Thus, to identify and verify differentially expressed miRNAs associated with embryo development potential, we chose candidate miRNAs in Group 1 with Raw Ct (miRNA) < 30 and ΔCt (miRNA) < 10 to exclude miRNAs with low expression levels. ΔCt = Raw Ct (miRNA) − Raw Ct (internal reference U6). The smallerΔCt is, the higher relative expression quantity the corresponding miRNA has. As shown in Table 2, 15 miRNAs (miR-222, miR-320, miR-24, miR-132, let-7b, miR-106a, miR-19b, miR-16, miR-186, miR-339-3p, miR-17, miR-323-3p, miR-197, miR-20a, and miR-382) were down-regulated in Group 2 and were chosen for subsequent verification analysis. These miRNAs had the highest relative expression quantities, and these decreased from Group 1 to Group 2 based on the miRNA profiling results. There were no up-regulated miRNAs in Group 2.

**Figure 2:**
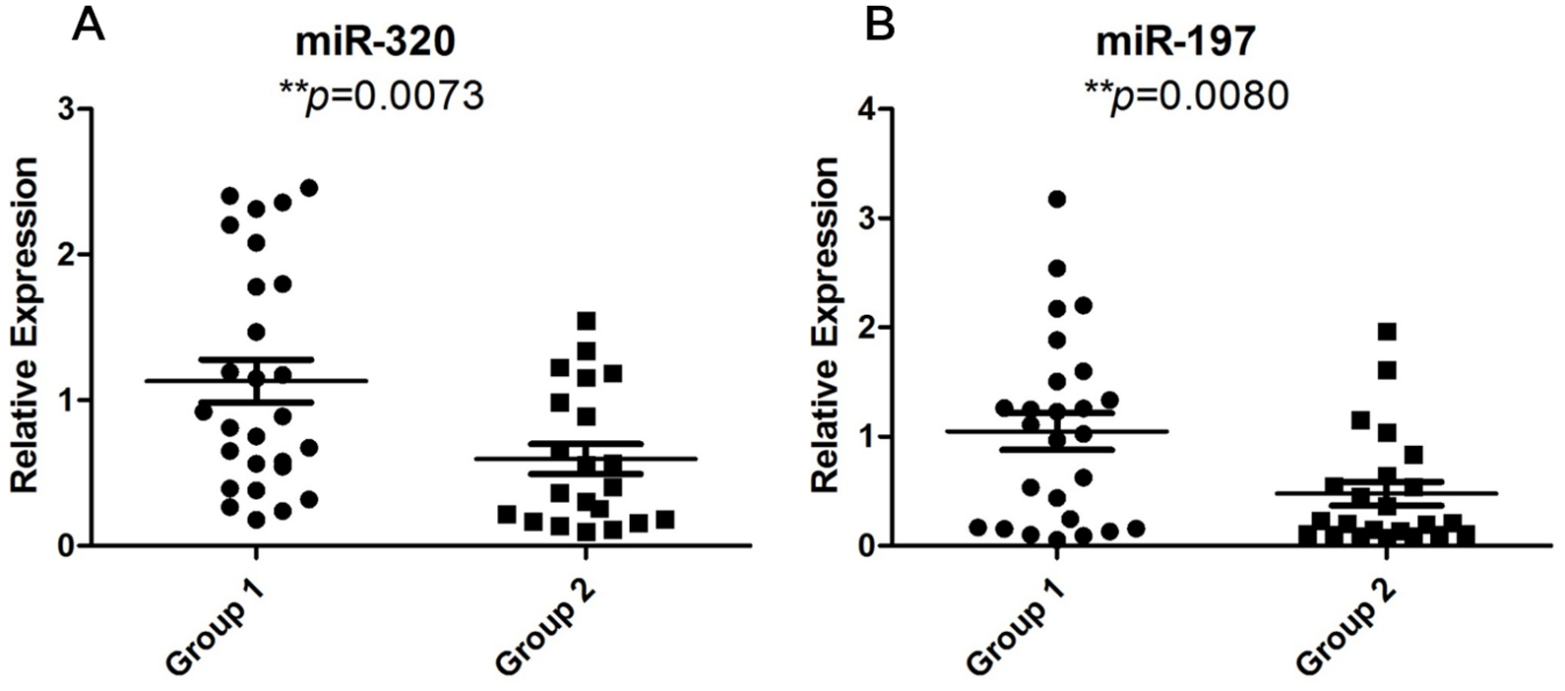
Significant expression levels of miRNAs. Scatter plots depicting significantly different levels of miR-320 (A) and miR-197 (B) between the two groups. Unpaired t-test, * represents *p* < 0.05; ** represents *p* < 0.01.

We measured the expression levels of these candidate miRNAs by qRT-PCR with Taqman miRNA assay in each follicular fluid sample in the two groups. As indicated in Figure 2, among the 15 candidate miRNAs, only miR-320 (p = 0.0073, Figure 2A) and miR-197 (p = 0.0080, Figure 2B) were found to be significantly different between the two groups. Other miRNAs were not associated with embryo quality (Supplementary Figure 1A and 1B).

### Knockdown of mmu-miR-320 in mouse MII oocytes affects embryonic development

**Figure 3:**
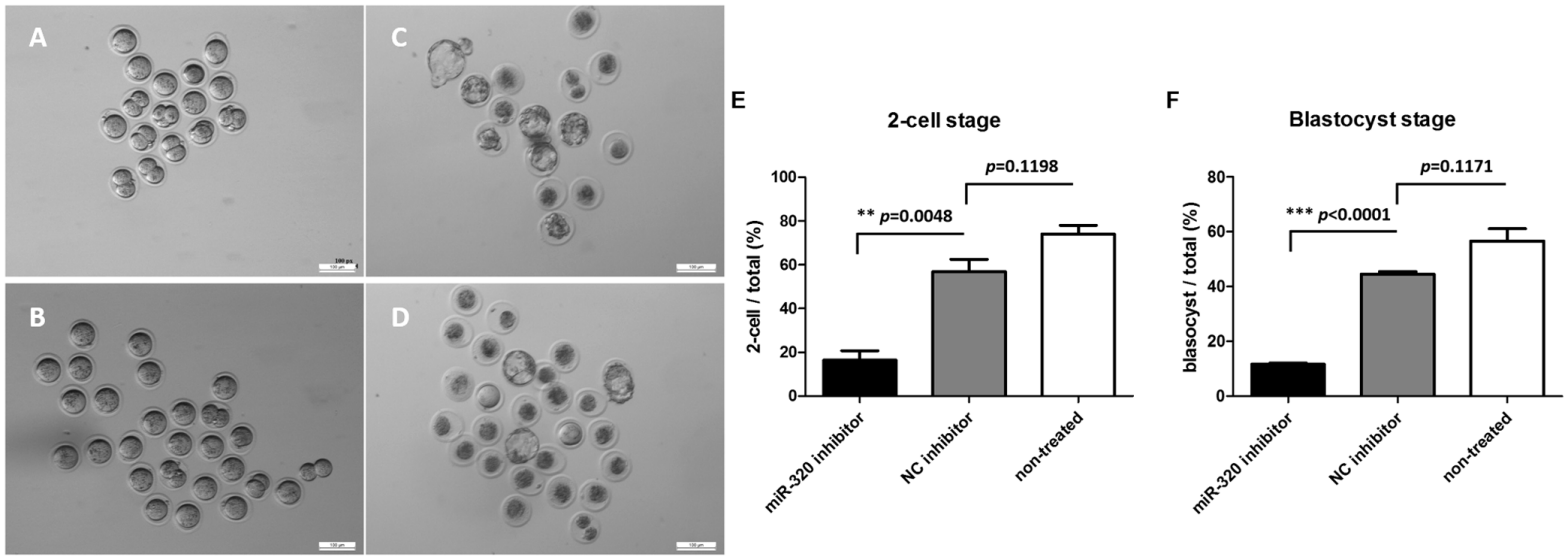
Morphology and statistical results of the 2-cell stage and blastocyst stage of oocytes injected with either miR-320 inhibitor or negative control (NC) inhibitor and oocytes of non-treated control group. Morphology of the 2-cell stage and blastocyst stage of miR-320 inhibitor-injected (n = 112), NC inhibitor-injected (n = 80) and non-treated (n = 180) *in vitro* fertilized mouse oocytes. The 2-cell stage of miR-320 inhibitor-injected oocytes (A) and NC inhibitor-injected oocytes (B). The blastocyst stage of miR-320 inhibitor-injected oocytes (C) and NC inhibitor-injected oocytes (D). The proportions of MII oocytes in the miR-320 inhibitor and NC inhibitor-injected groups that developed into the 2-cell stage (E) were 16.41% ± 4.33%, 56.85% ± 5.71% and 75% ± 7.07%, respectively. The proportions of MII oocytes in the miR-320 inhibitor and NC inhibitor-injected groups that developed into the blastocyst stage (F) were 11.70% ± 0.42%, 39.26% ± 5.37% and 56% ± 5.66%, respectively. Injection experiments were performed in triplicate. Unpaired t-test, * represents p < 0.05; ** represents p < 0.01, *** represents p < 0.001. NC, negative control.

To further investigate the function of miRNAs in affecting embryonic developmental potential, mouse oocyte/embryonic development was evaluated by knocking down the miRNAs with their corresponding miRNA inhibitors. Because there is no miR-197 or homologous miRNA in the mouse miRNA database, our in vitro experiments focused solely on studying the function of miR-320. Supplementary Figure 2 shows that miR-320 expression was strongly reduced after injection of its inhibitor. In the negative control (NC) group, oocytes were injected with universal oligonucleotides provided by the manufacturer which is not homologous with any known mammal genes in the same dosage with miR-320 inhibitor. There was also a blank control group with non-treated oocytes. After cultivation for 8 hours, these MII oocytes were fertilized in vitro and embryo development was evaluated at the 2-cell stage and the blastocyst stage. As Figure 3 shows, mouse embryo development in the miR-320 inhibitor-injected group was significantly affected. The proportions of MII oocytes in the miR-320 inhibitor-injected group that developed into 2-cell stage and blastocyst-stage embryos were 16.41% ± 4.33% and 11.70% ± 0.42%, respectively (n = 112). In the NC group, the proportions of MII oocytes that developed into 2-cell stage and blastocyst-stage embryos were 56.85% ± 5.71% and 39.26% ± 5.37%, respectively (n = 80). In the non-treated group, the proportions of MII oocytes that developed into 2-cell stage and blastocyst-stage embryos were 75% ± 7.07% and 56% ± 5.66%, respectively (n = 180). Considering the physical damage, the proportion to 2-cell stage and blastocyst-stage were lower than those in the non-treated group without reaching a significant level, which we thought acceptable.

### Expression levels of Wnt signaling pathway components were abnormal in miR-320 inhibitor-injected oocytes

**Figure 4:**
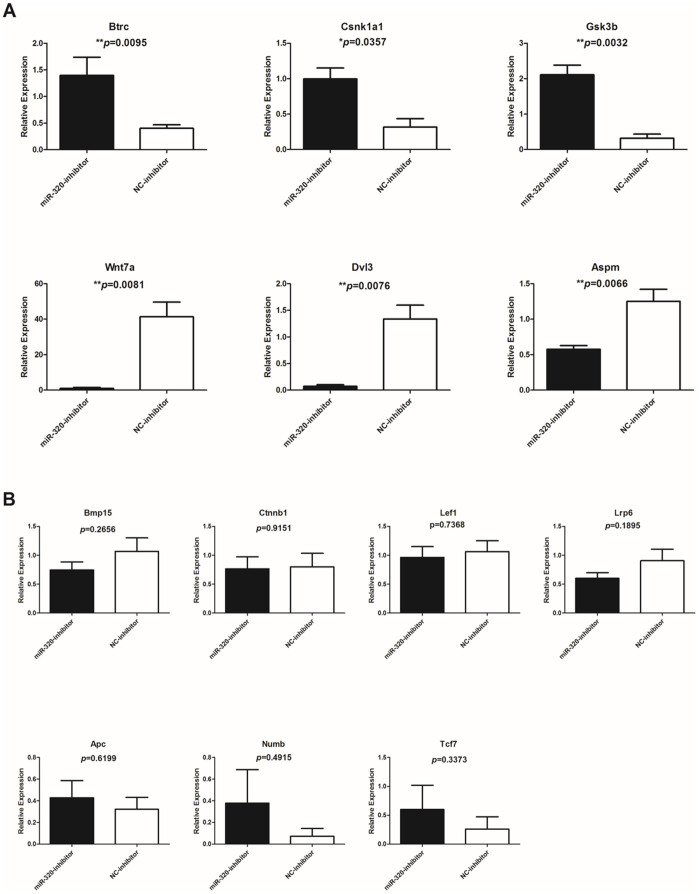
Significant and insignificant expression of Wnt signaling pathway genes in oocytes injected with either miR-320 inhibitor or NC inhibitor. Histograms depicting six significantly different gene expression (A) and seven insignificant gene expression (B) within or regulate Wnt signaling pathway between miR-320 inhibitor-injected (n = 80) and NC inhibitor-injected (n = 50) groups. Injection experiments were performed in triplicate. Unpaired t-test, * represents p < 0.05; ** represents p < 0.01. NC, negative control.

Several studies demonstrated that Wnt signaling pathway plays an important role in fertilization and embryo development^21^,^22^,^23^,^24^. Hsieh et al. have indicated that Wnt signal pathway could be regulated by miR-320^25^. To investigate the mechanisms behind the impaired fertilization and development competence of miR-320 inhibitor-injected oocytes, we tested 13 genes of Wnt signaling pathway to see if there were any abnormal expression levels that might affect the oocyte (Figure 4A and 4B). Three genes – Btrc, Csnk1a1 and Gsk3b were significantly increased compared to the control group and other three genes – Wnt7a, Dvl3 and Aspm were significantly decreased compared to the control group (Figure 4A). Another seven genes – Bmp15, Ctnnb1, Lef1, Lrp6, Apc, Numb and Tcf7 were found to have no significant difference between the miR-320 inhibitor-injected (n = 80) and NC inhibitor-injected (n = 50) groups (Figure 4B).

## Discussion

In this study, we collected follicular fluid from the first (the largest) follicles of 68 patients who underwent ICSI treatment. Altogether, 53 samples of follicular fluid containing MII oocytes were analyzed for oocyte development outcomes after fertilization. We classified follicular fluid samples into two groups according to the embryo qualities on the third day after fertilization. MiRNA profiles of each group were determined, and miR-320 and miR-197 were found to have significantly different expression levels between the two groups. We subsequently found that the proportions of mouse MII oocytes that developed into 2-cell and blastocyst-stage embryos were strongly affected by knockdown of miR-320, and this further indicated that miR-320 plays an important role in embryo development potential. We also measured gene expression of Wnt signaling pathway and found abnormally increased and decreased genes that might negatively affect oocyte competence for fertilization and early development. Thus, we have provided the first report on miRNAs in human follicular fluid being associated with embryo quality both in vivo and in vitro.

Follicular fluid is a complex mixture of various kinds of proteins, hormones, vitamins, cytokines, and metabolites, and numerous studies have demonstrated that the ingredients of the follicular fluid influence the development of reproductive disorders. For example, one study showed that coagulation factors in the follicular fluid are associated with recurrent spontaneous abortion^26^. In addition, leptin levels in the follicular fluid of PCOS patients are significantly higher than those in controls^27^,^28^.

ART have been applied in clinical practice for more than thirty years. Although the number of IVF cycles being performed increases every year, only about 30% of IVF cycles currently result in a pregnancy^3^,^29^. Embryo quality is a key factor for successful pregnancy, and morphological characteristics and cleavage rates are currently the main clinical criteria for selecting and transferring embryos^30^,^31^. Because the follicular fluid provides the microenvironment for oocyte development, investigators are trying to identify predictive biomarkers in the follicular fluid that are associated with fertility-related phenotypes, including follicle development, oocyte fertilization, embryo quality, and pregnancy outcomes. For example, the concentration of anti-Müllerian hormone in follicular fluid was found to be a predictive variable of the implantation potential of IVF-obtained oocytes^32^. A negative association was found between homocysteine concentrations in the follicular fluid and oocyte/embryo qualities in PCOS patients^33^. In addition, a relationship between the metabolic profile of the follicular fluid and oocyte developmental potential and implantation outcome has been uncovered^11^,^12^. During follicular development, one follicle contains one oocyte so the quality of the oocyte and its outcome after fertilization should be reflected by the ingredients of the matching follicular fluid. However, most previous studies collected and pooled follicular fluid from multiple follicles. Moreover, in those studies IVF, not ICSI, methods were used. Thus, fertilization outcomes cannot be evaluated individually in the previous studies and the identified ingredients in the follicular fluid might not be truly reflective of oocyte/embryo quality.

In our previous study, we identified for the first time the existence of miRNAs in human follicular fluid^18^. We found some miRNAs that regulated steroidogenesis in vitro and some that were associated with PCOS in vivo^18^. Recently, Diez-fraile et al. demonstrated that age-associated differential microRNA levels in human follicular fluids may reveal pathways potentially determining fertility and success of in vitro fertilization^19^. In addition, Santonocito et al. identified 37 microRNAs upregulated in follicular fluids as compared with plasma from the same women^20^. Bioinformatics analysis reveals these microRNAs are involved in important pathways for follicle growth and oocyte maturation. They suggest these microRNAs may represent noninvasive molecular markers of oocyte quality in ART^20^. However, until now, there is still no study reporting relationship between microRNAs in follicular fluids and embryo development. Thus, in the present study, we hypothesized that there might be miRNAs in the follicular fluid that are associated with embryonic development potential. To evaluate the relationship between follicular fluid contents and oocyte/embryo development, it is imperative that follicles should be aspirated individually. Thus only the fluid of the first follicle was collected from each ICSI patient to guarantee that each sample of follicular fluid was accurately matched to the very first follicle and the first retrieved oocyte of each patient. By miRNA profiling and qRT-PCR, we identified miR-320 and miR-197 in the follicular fluid as potential candidates for being associated with embryo development potential. Both miRNAs have higher expression levels in the Group 1, which consists of MII oocytes that can develop into top-quality embryos.

To further investigate the role of miR-320 in embryonic development, we knocked down its expression in mouse MII oocytes by injecting its inhibitor oligonucleotide. Most of the miR-320 inhibitor-injected embryos arrested at the 2-cell stage and only a few proceeded to develop into blastocysts indicating that miR-320 is essential for embryonic development. Thus the association between the miR-320 expression level in follicular fluid and embryonic development was supported in ICSI patients as well as the in vitro fertilization and cultivation of mouse oocytes.

Diez-fraile et al. demonstrate that miR-320 were among the top 10 highest expressed miRNAs in follicular fluid^19^, while Santonocito et al. did not identify miRNA-320 in follicular fluid^20^. There are some possible explanation for the inconsistency. First, different ethnic populations have genetic heterogeneity, which may result in different expression of genetic ingredient in body fluids. Second, patients may adopt different stimulation protocol, which may cause expression changes of genes or microRNAs.

MiRNAs have been well studied for nearly two decades^34^, and they have been shown to be involved in numerous biochemical processes^35^, pathological mechanisms^36^,^37^, and evolutionary processes^38^. Although some reports show that miRNA function is suppressed in oocytes^39^,^40^, mouse oocytes missing the miRNA-processing enzyme Dicer do not have miRNAs and exhibit disorganized spindles, and embryos deriving from these Dicer-deficient oocytes cannot pass through the first cleavage^41^. In addition, miR-335 was found to play an important role in oocyte meiotic maturation^42^ and miR-135A regulates early embryonic development^43^. These pieces of evidence indicate that maternal microRNAs are essential for oocyte and early embryonic development.

It has been shown that miRNAs play key roles in a number of signaling pathways^14^, and there is evidence to suggest that miR-320 participates in regulating multiple signaling pathways, including Wnt signaling and insulin–PI3K signaling^25^,^44^. Wnt signaling pathway regulates a large variety of cellular processes including cell proliferation, cell-cell adhesion, cell fate decision, pluripotency and polarity establishment^21^,^45^,^46^. Components of Wnt signaling pathway are widely expressed in mouse ovaries, oocytes, and cleavage-stage embryos^47^, and there are articles reported that Wnt signaling pathway is essential in preimplantation embryo development^21^,^23^,^48^ and ovarian follicle development^49^,^50^. Takezawa et al. have indicated that the member of Wnt signaling pathway is involved in cell membrane adhesion and fusion during fertilization^51^. Though lack of the center molecule β-catenin did not affect blastocyst formation^21^,^45^, there is growing evidence indicating that canonical Wnt/β-catenin signaling pathway regulates embryo development to the blastocyst stage. Lim et al. found that specific inhibition of Wnt/β-catenin signaling pathway would promote pig blastocyst hatching, increase total and trophectoderm cell number; vice versa^52^. Denicol et al. showed that activation of canonical Wnt signaling pathway after the major zygote genome activation would decrease the bovine embryo development to blastocyst and reduce cell numbers of inner cell mass and trophectoderm^48^. They also demonstrated in a separate paper that the WNT signaling antagonist Dickkopf-1 could improve embryo survival after transfer to recipients, which shed light upon the application to improvement of assisted reproductive technologies^22^. Furthermore, Xie et al. reported inhibition of canonical Wnt signaling pathway would notably block the blastocyst competence to implantation^23^. In addition, Hsieh et al. have indicated that Wnt signaling pathway could be regulated by miR-320^25^. Altogether, these imply that Wnt signaling pathway might have effects in fertilization and early embryo development and it may be altered by knocking down the level of miR-320.

Therefore, we tested 13 genes related to Wnt signaling pathway in treated mouse oocyte. We found abnormal expression of *Btrc*, *Csnk1a1*, *Gsk3b*, *Wnt7a*, *Dvl3* and *Aspm* in miR-320 inhibitor injected group. The ligand, *Wnt7a* and the intracellular molecule *Dvl3*, were significantly decreased in the miR-320 inhibitor-injected, while other three genes as members of β-catenin degradation complex – *Btrc*, *Csnk1a1* and *Gsk3b* were significantly increased in the miR-320 inhibitor-injected group. Thus, these results altogether reflect inhibition of Wnt signaling pathway activity. Among the six differentially expressed genes, four of them (*Btrc*, *Csnk1a1*, *Gsk3b* and *Aspm*) have been demonstrated to be essential in oocyte maturation, fertilization, and early development^53^,^54^,^55^,^56^,^57^,^58^,^59^.

*Btrc* encodes the E3 ubiquitin protein ligase and is a key mediator of meiotic arrest of MII oocytes and fertilization^53^. *Csnk1a1* encodes casein kinase 1 alpha (CK1α), and recent evidence demonstrates that CK1α regulates chromosome congression and separation during mouse oocyte meiotic maturation and early embryo development^54^. The expression of both CK1α mRNA and protein are slightly decreased and continue to decrease in early 1-cell embryos^54^. However, in our study, the expression of *Csnk1a1* were significantly increased in miR-320 inhibitor-injected group, which resembled the phenotypes of treatment of pyrvinium pamoate in MI oocyte^54^ and therefore, may result in impairment of spindle structure and chromosome alignment and compromise its function during fertilization and embryo development. For *Gsk3b*, the evidence suggests that it regulates chromatin segregation and cytokinesis in mouse preimplantation embryos^55^. Similar to *Ck1α*, the expression level of both Gsk3b mRNA and protein are relatively low in MII oocytes and decrease in 1-cell embryos^55^,^56^,^57^, while it had significantly higher expression levels in miR-320 inhibitor-injected oocytes. Besides, Harris et al. reported that the inhibition of *Gsk3b* from zygote stage would increase the cell number of blastocyst^57^. It is conceivable that the significant higher level of *Gsk3b* may have the opposite effect to reduce the embryo cell number after we injected the oocytes with miR-320 inhibitor, or further more might influence the Day 3 embryo cell number of our ICSI patients whose follilular fluids contained relatively low level of miR-320. *Aspm*, as reported as a positive regulator of Wnt signaling pathway^59^, were down-regulated in the miR-320 inhibitor-injected group. Down regulation of *Aspm* expression was reported to disrupt meiotic spindle organization in mouse oocytes^58^, which may indicate that the low level of *Aspm* in miR-320 inhibitor-injected group may contribute to the damaged capacity of oocytes to fertilize and develop to 2-cell and blastocyst stage. Taken together, abnormal expression of Wnt signaling pathway related genes might contribute to the decreased 2-cell rate and blastocyst rate in the miR-320 inhibitor-injected oocytes.

In conclusion, we have found that miR-320 and miR-197 in human follicular fluid are associated with embryonic development potential. Knocking down miR-320 in mouse oocytes negatively affected embryonic developmental potential by inhibiting expression of Wnt signaling pathway. This study provides the first piece of evidence that miRNAs in human follicular fluid might reflect and affect embryo quality. These results increase our understanding of molecules in the human follicular fluid that are related to embryo quality and lay the foundation for the future development of miRNAs in the follicular fluid as novel biomarkers for embryo quality and other fertility-related phenotypes.

## Methods

### Subjects

Sixty-eight infertile women were included in this study. They were undergoing ICSI treatment for male factor or female tubal factor infertility in Shanghai Ji Ai Genetics and IVF Institute affiliated with Fudan University from January 2013 to August 2013. Their ages ranged from 23 years to 49 years with an average age of 34 years. Body mass index (BMI) ranged from 18.1 kg·m−2 to 30.1 kg·m−2 with an average value of 22.0 kg·m−2. After removing immature and other abnormal oocytes, 53 samples were used in the experiment. The use of follicular fluid obtained during oocyte pick-up for the ICSI process was allowed by the patients with written informed consent. This research was approved by the Institutional Review Committee of Fudan University. All experiments were performed in accordance with relevant guidelines and regulations.

### Ovarian stimulation and cycle monitoring

Patients were stimulated with recombinant FSH (rFSH, Serono, Geneva, Switzerland) after being down-regulated with GnRH agonists (GnRH-a, Serono, Geneva, Switzerland) according to the long protocol. Follicular development was monitored by real-time ultrasound scans and serum hormone levels. When at least one side of the ovarian follicle reached 18 mm in diameter, 10,000 IU of hCG (Livzon Pharmaceutical Group, Zhuhai, China) was administered 34–36 hours prior to follicular puncture.

### Follicular fluid sampling and oocyte collection

Follicular fluid was obtained by trans-vaginal ultrasound-guided puncture and aspiration of >10 mm diameter follicles. Follicular fluid from the first aspirated follicle of each patient was collected carefully and centrifuged at 3865× g for 5 minutes^17^. The supernatant was collected and the centrifugation was repeated to completely remove cellular fragments and blood contents, and the sample was frozen at −80°C until the RNA extraction. Typically, 4–6 mL of follicular fluid were collected from the very first aspirated follicle of each patient. The cumulus-oocyte complex obtained from the corresponding follicular fluid was carefully isolated. Hyaluronidase digestion was performed to remove cumulus cells surrounding the oocyte. If the oocyte was immature (GV or MI) or in atresia, then ICSI treatment of this oocyte was stopped. Mature oocytes (MII) continued to receive the ICSI treatment. We only included one oocyte from each patient in this study.

### Evaluation of embryo grading

After ICSI, oocytes were cultured in a humid atmosphere of 5% O2 and 5% CO2 at 37°C. Fertilization occurred within 14–16 hours after the ICSI treatment. If two pronuclei were observed, then the oocyte was considered normal. If no pronuclei were observed, then the oocyte was regarded as a failure. If any other number of pronuclei were present, the oocyte was considered the result of abnormal fertilization. Only normal fertilized oocytes continued to be cultured, and embryo quality was assessed three days after fertilization. We combined cell number and degree of cytoplasmic fragmentation to grade embryo quality and competence^60^,^61^. Group 1 consisted of high-quality embryos that contained seven or more cells, and Group 2 consisted of lower-quality embryos that contained six or fewer cells. Only Group 1 embryos would be considered in subsequent embryo transplantation treatment. In the present study, Group 1 embryos were transferred back to the patients as part of the routine of the ICSI treatment or were frozen in liquid nitrogen according to the patient's decision. Group 2 embryos were not used for transplantation nor were they worth preserving, and these were discarded with the patient's consent.

### RNA extraction, TaqMan® miRNA array, and miRNA profiling

Based on the oocyte grading described above, 29 follicular fluid samples were classified as Group 1 and 24 samples were classified as Group 2. The samples in the two groups were pooled separately, and the RNA samples from the pooled follicular fluid were used for miRNA expression profiling with the TaqMan® Array Human MicroRNA A + B Cards Set v3.0 (Applied Biosystems, Foster City, CA, USA). The methods were in accordance with our previous publication^18^. The total RNA was extracted with the miRNeasy Kit (QIAGEN, Hilden, Germany), and the same amount of RNA was used in the reverse-transcription experiments with the TaqMan miRNA assay. The expression levels of individual miRNAs, including hsa-miRNA-320 and hsa-miRNA-197, were detected by corresponding TaqMan® miRNA assays (Applied Biosystems) in each of the 53 samples and were normalized by comparison to the internal reference U6 snRNA^18^,^62^,^63^. The relative expression level equals 2−ΔCt where ΔCt = Ct (miRNA) − Ct (U6).

RNA extraction, TaqMan® miRNA array, and miRNA profiling methods were adapted from those described in our previous study^18^. Briefly, total RNA was extracted with the miRNeasy Kit (QIAGEN, Hilden, Germany) according to the manufacturer's directions. A total of 500 μL of follicular fluid supernatant from each patient was placed in a 15 mL AxygenTM centrifuge tube (Corning, Tewksbury, MA, USA) and thoroughly mixed with 2.5 mL of QIAzol Lysis Reagent (QIAGEN, Hilden, Germany). The mixture was incubated at room temperature for 5 minutes and 500 μL of chloroform was added and vortexed vigorously. The samples were centrifuged at 11,000 rpm for 30 minutes at 4°C. The upper aqueous phase was transferred carefully to a new 15 mL centrifuge tube, and 1.5 volumes (usually 5 mL) of absolute ethyl alcohol were added. The mixtures were applied to RNA binding columns and washed twice. Total RNA was eluted in 30 μL of nuclease-free H2O.

### Mouse oocyte collection, microinjection, and in vitro fertilization

Six-week-old female B6D2F1 mice were superovulated by intraperitoneal injection of pregnant mare serum gonadotropin (PMSG, 5 IU per mouse, Ningbo Second Hormone Factory, Ningbo, China) followed by human chorionic gonadotropin 48 hours later (hCG, 5 IU per mouse, Ningbo Second Hormone Factory, Ningbo, China). MII oocytes were collected from oviduct ampullae 13–15 hours post hCG injection. Cumulus cells were removed after a 2 minute digestion in 300 IU·mL−1 hyaluronidase in M2 medium (Sigma-Aldrich, St. Louis, MO, USA). Denuded oocytes were washed in M2 medium and kept at 37°C in 5% CO2 until injection.

Microinjection of the miR-320 inhibitor was performed in M2 medium using a Leica Hoffman microscope (LSM6000) equipped with the TransferMan NK2 micromanipulator and InjectMan NI2 (Eppendorf, Hamburg, Germany). A total of 5–10 pL of the miR-320 inhibitor (50 μmol·L−1) was injected into the cytoplasm of MII oocytes. The same volume of negative control (NC) inhibitor (50 μmol·L−1) was injected into control oocytes. The negative control were provided by the manufacturer, which are universal oligonucleotides not homologous with any known mammal genes. Inhibitor oligonucleotides were synthesized by GenePharma, Shanghai, China. About 50 oocytes were injected each time, and each injection experiment was repeated at least three times. After injection, oocytes were cultured in M2 medium for 8 hours and then used for IVF. The MII oocytes microinjected with miR-320 inhibitor and its NC inhibitor were placed in 500 μL EmbryoMax® Human Tubal Fluid (HTF, Millipore, Billerica, MA, USA) medium in one well of a 4-well plate under mineral oil. A total of 100 μL spermatozoa (final concentration 10,000–20,000 spermatozoa·mL−1) that had previously been capacitated for 1 hour was added. Co-incubation was carried out for at least 5 hours at 37°C in 5% CO2 under mineral oil to prevent evaporation and pH changes. After this time, fertilized and unfertilized oocytes were cultured in EmbryoMax® KSOM (Millipore, Billerica, MA, USA) media. The 2-cell formation rate and blastocyst rate were recorded at day 2 and day 4 post-fertilization. Institutional Review Committee of Fudan University approved animal protocol.

### Expression of Wnt signaling pathway genes in miR-320 inhibitor-injected and control groups

We collected oocytes 8 hours (just before insemination) after injection with miR-320 inhibitor (n = 80) or NC inhibitor (n = 50) and measured the expression levels of 13 genes (Apc, Aspm, Btrc, Csnk1a1, Ctnna1, Ctnnb1, Dvl3, Gsk3b, Lef1, Lrp6, Numb, Tcf7 and Wnt7a) within or regulate Wnt signaling pathway by qRT-PCR. Prior to the PCR, whole transcriptome amplification (TaKaRa, Dalian, China) was performed due to the limited quantity of RNA in the small numbers of oocytes. Experiments were performed in triplicate.

### Statistical analysis

Data in this study are reported as means of at least three independent experiments ± SEM unless otherwise noted. Student's t-test was performed to determine the significance of any difference in the miRNA expression levels between the two groups. A p-value <0.05 was considered statistically significant. All statistical analyses were carried out using SPSS software (version 16.0).

## Author Contributions

R.F., W.F., X.S., L.W., H.S., L.J. and L.H. conceived and designed the experiments. W.F., Y.a.X., X.S., Q.S., Y.o.X., R.C. and R.Q. collected the samples. R.F., Y.Z., M.L. and Y.o.X. performed the experiments. R.F., Y.a.X. and L.W. analyzed the data. R.F., Y.Z. and L.W. wrote the paper. All authors reviewed the manuscript.

## Supplementary Material

Supplementary Information Supplementary Figures and Tables

## Figures and Tables

**Table 1:** Clinical characteristic of subjects undergoing ICSI, graded by embryo status three days after fertilization

	Group 1 (n = 29)	Group 2 (n = 24)	p-values
**Ages (years)**	35.4 ± 6.0	32.9 ± 5.5	0.1078
**BMI (kg·m^−2^)**	22.2 ± 3.0	21.9 ± 2.8	0.7982
**Months of infertility**	61.1 ± 31.3	74.4 ± 44.1	0.2075
**Cycle length (days)**	30.4 ± 5.3	33.5 ± 11.9	0.1839
**Day-3 E2 (pg·mL^−1^)**	35.8 ± 16.3	36.6 ± 21.0	0.9047
**Day-3 P4 (ng·mL^−1^)**	0.8 ± 1.0	0.7 ± 0.4	0.7738
**Day-3 LH (mIU·mL^−1^)**	5.5 ± 4.1	4.6 ± 3.7	0.4569
**Day-3 FSH (mIU·mL^−1^)**	8.8 ± 3.3	7.5 ± 2.2	0.1155
**E2 (pg·mL^−1^) of OPU**	3160.0 ± 1685.1	3642.8 ± 1327.7	0.2979
**P4 (ng·mL^−1^) of OPU**	1.5 ± 1.3	1.4 ± 0.7	0.7705
**LH (mIU·mL^−1^) of OPU**	8.7 ± 12.1	5.5 ± 4.7	0.1609
**Number of >10mm follicles**	9.3 ± 3.4	11.2 ± 4.8	0.1143
**Number of oocytes**	8.3 ± 4.3	9.7 ± 4.7	0.3071
**Number of mature oocytes**	6.8 ± 3.5	7.9 ± 3.9	0.3154
**Number of fertilized embryos**	5.8 ± 2.8	6.8 ± 4.0	0.3131
**Number of cleaved embryos**	5.6 ± 2.9	6.5 ± 3.9	0.3651
**Number of available embryos**	4.3 ± 2.9	4.1 ± 2.9	0.8686

Data are mean ± SD. Group 1: 7 or more cells; Group 2: 6 or fewer cells. P-values: Unpaired t test. BMI, body mass index; E2, estradiol; P4, progesterone; LH, luteinizing hormone; FSH, follicle stimulating hormone; OPU, oocyte pick up.

**Table 2:** MicroRNAs with high expression level and that change with the same tendency as embryo quality between the two groups

	Group 1			Group 2		
	Raw Ct	ΔCt	RQ	Raw Ct	ΔCt	RQ
**hsa-miR-222**	22.9353	3.4933	88.7998	29.9372	8.0152	3.8653
**hsa-miR-320**	22.956	3.514	87.5348	25.9736	6.1246	14.3322
**hsa-miR-24**	23.979	4.537	43.0752	26.9352	7.0862	7.3594
**hsa-miR-132**	23.9837	4.5417	42.9351	29.9722	8.0152	3.8653
**hsa-let-7b**	26.0723	6.6303	10.0944	27.9555	8.0195	3.8538
**hsa-miR-106a**	26.9474	7.5054	5.5036	30.9716	11.1226	0.4485
**hsa-miR-19b**	26.9589	7.5169	5.4599	30.0096	10.1606	0.8737
**hsa-miR-16**	26.9602	7.5182	5.4550	30.9374	11.0884	0.4593
**hsa-miR-186**	27.9437	8.5017	2.7588	31.9973	12.1483	0.2203
**hsa-miR-339-3p**	27.9707	8.5287	2.7077	31.9617	12.1127	0.2258
**hsa-miR-17**	27.9708	8.5288	2.7075	31.014	11.165	0.4355
**hsa-miR-323-3p**	28.9357	9.4937	1.3871	31.9726	12.1236	0.2241
**hsa-miR-197**	28.9479	9.5059	1.3754	29.8681	10.0191	0.9637
**hsa-miR-20a**	28.9754	9.5334	1.3494	33.0135	13.1645	0.1089
**hsa-miR-382**	29.0083	9.5663	1.3190	32.0059	12.1569	0.2190

Internal reference: snRNA U6. ΔCt = Raw Ct (miRNA) − Raw Ct (U6). Inclusion criteria: Raw Ct (Group 1) < 30, ΔCt (Group 1) < 10, and ΔCt (Group 1) < ΔCt (Group 2). RQ, relative quantity, RQ = 2 ^(−ΔCt)^*1000.
