# Supplementary material for: MiRNA-320 in the human follicular fluid is associated with embryo quality in vivo and affects mouse embryonic development in vitro
Source: Sci Rep. 2015 Mar 3;5:8689. doi: 10.1038/srep08689 (PMC4346788; doi:10.1038/srep08689)
Supplement: Supplementary Information — Supplementary Figures and Tables [file srep08689-s1.doc]

MiRNA-320 in the human follicular fluid is associated with embryo quality *in vivo* and affects mouse embryonic development *in vitro*

Ruizhi Feng^1,2,¶^, Qing Sang^1,2¶^,Yan Zhu^4,¶^,Wei Fu^3,¶^, Miao Liu^5^, Yan Xu^3^, Huijuan Shi^5^, Yao Xu^1,2^, Ronggui Qu^1,2^, Renjie Chai^6^, Li Jin^1^, Lin He^2,7^, Xiaoxi Sun^3*^ and Lei Wang^1,2*^

**Supplementary Information**

**Supplementary Figure 1. Insignificant miRNAs between Group 1 and Group 2.** Scatter plots depicting the levels of 13 miRNAs (A) and (B) between the two groups. Unpaired t-test.


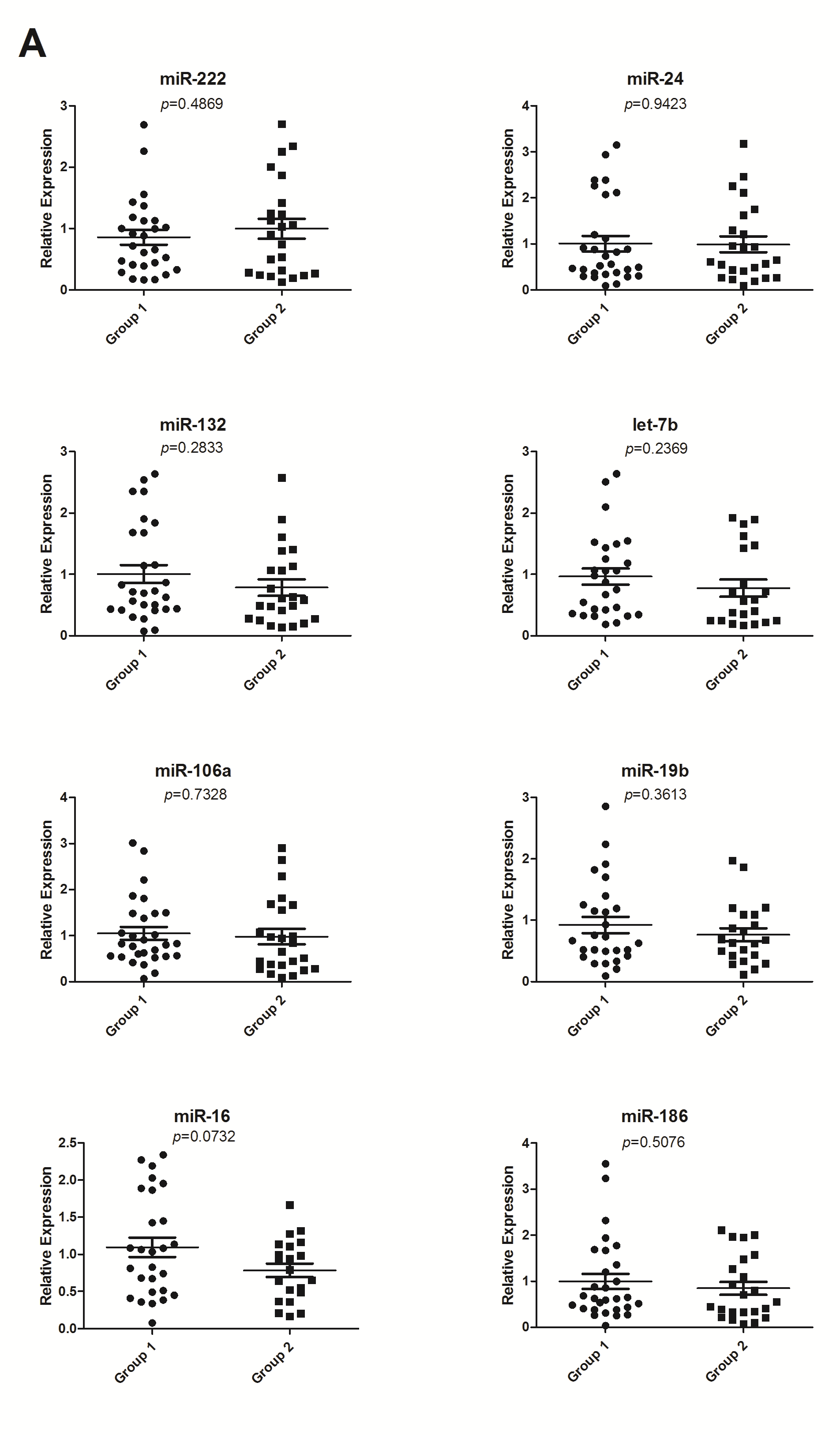


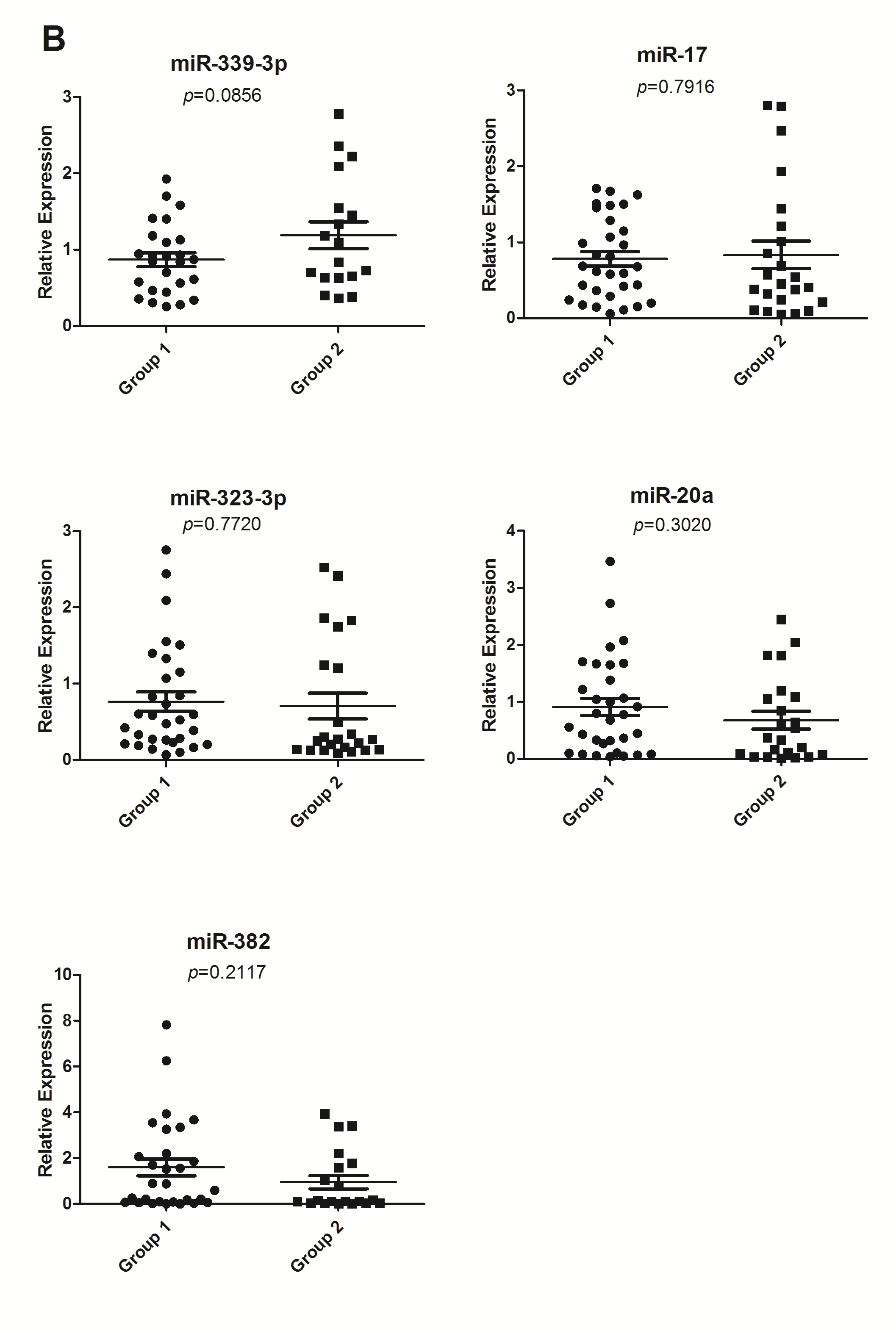


**Supplementary Figure 2. Reduced miR-320 expression level in miR-320 inhibitor-injected group compared with NC-injected group.** Relative expression level of miR-320 in the miR-320 inhibitor-injected group and NC inhibitor-injected group as detected by TaqMan® miRNA assay verifying the effectiveness of the knockdown experiment. NC, negative control.


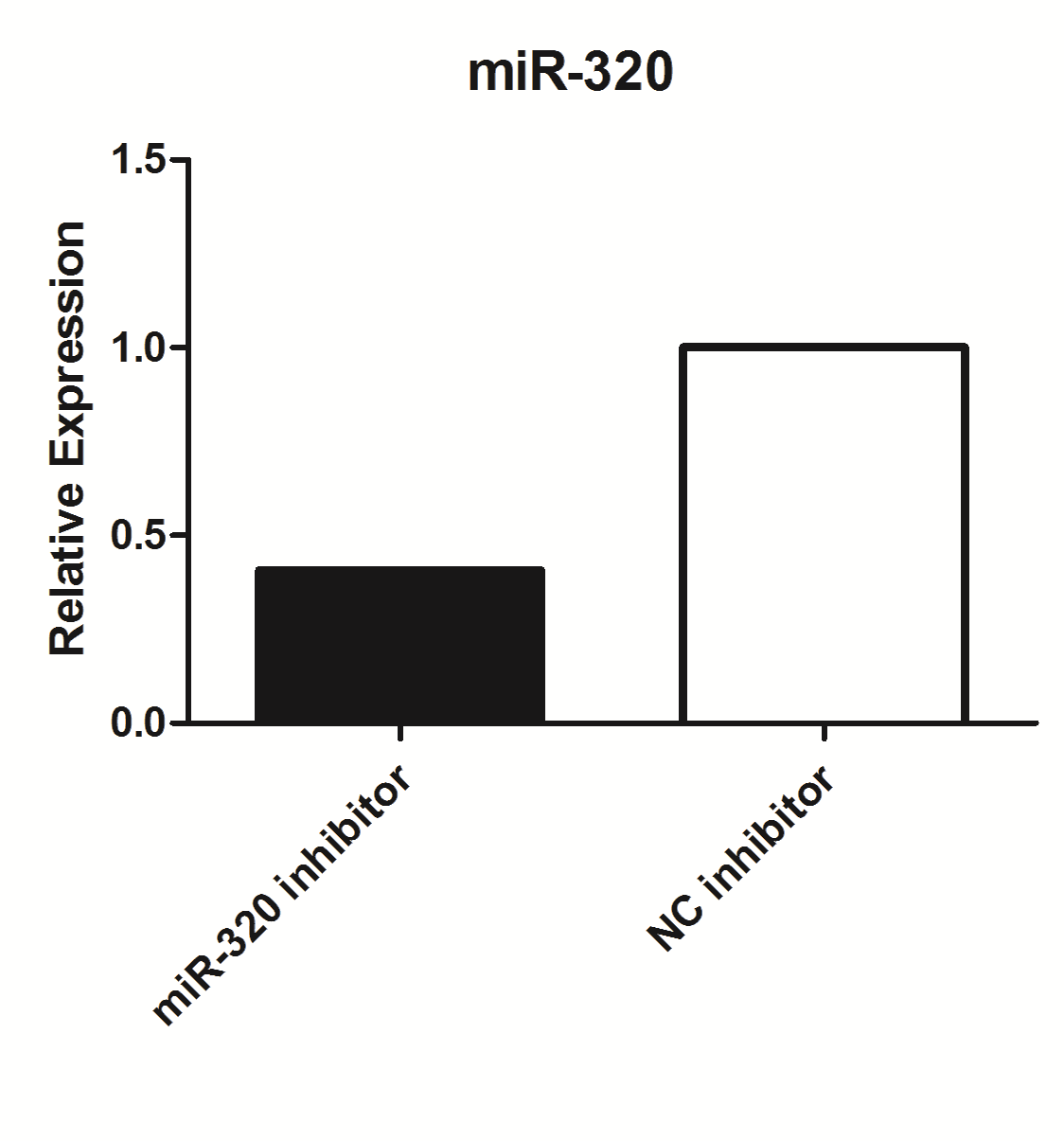


**Supplementary Table 1. microRNAs with high expression level (Raw Ct <30) in two groups** of pooled follicular fluid samples.

| **Group 1** | **Raw Ct** | **Group 2** | **Raw Ct** |
| --- | --- | --- | --- |
| hsa-miR-1243 | 22.0565 | hsa-miR-193b | 21.9614 |
| hsa-miR-222 | 22.9353 | hsa-miR-222 | 22.9375 |
| hsa-miR-193b | 22.9502 | hsa-miR-320 | 22.9406 |
| hsa-miR-320 | 22.9560 | hsa-miR-574-3p | 22.9606 |
| hsa-miR-628-5p | 23.9191 | hsa-miR-1274B | 22.9872 |
| hsa-miR-24 | 23.9790 | hsa-miR-191 | 23.9618 |
| hsa-miR-132 | 23.9837 | hsa-miR-24 | 23.9797 |
| hsa-miR-574-3p | 23.9889 | hsa-miR-132 | 23.9804 |
| hsa-miR-191 | 24.9684 | hsa-miR-720 | 23.9888 |
| hsa-miR-1274B | 24.9911 | hsa-miR-484 | 24.9624 |
| hsa-miR-1290 | 25.9592 | hsa-miR-223 | 24.9632 |
| hsa-miR-223 | 25.9659 | hsa-miR-1290 | 24.9689 |
| hsa-let-7b | 26.0723 | hsa-miR-1233 | 24.9691 |
| hsa-miR-30a-5p | 26.9429 | hsa-miR-146a | 24.9782 |
| hsa-miR-106a | 26.9474 | hsa-miR-30a-5p | 25.8823 |
| hsa-miR-19b | 26.9589 | hsa-miR-212 | 25.9199 |
| hsa-miR-720 | 26.9602 | hsa-let-7b | 25.9715 |
| hsa-miR-16 | 26.9602 | hsa-miR-1260 | 25.9766 |
| mmu-miR-134 | 26.9835 | mmu-miR-134 | 26.0003 |
| hsa-miR-1233 | 27.0084 | hsa-miR-409-3p | 26.9558 |
| hsa-miR-186 | 27.9437 | hsa-miR-1274A | 26.9597 |
| hsa-miR-339-3p | 27.9707 | hsa-miR-150 | 26.9666 |
| hsa-miR-17 | 27.9708 | hsa-miR-16 | 26.9690 |
| hsa-miR-1274A | 27.9758 | hsa-miR-19b | 26.9728 |
| hsa-miR-409-3p | 27.9793 | hsa-miR-210 | 26.9992 |
| hsa-miR-663B | 28.9267 | hsa-miR-342-3p | 27.9068 |
| hsa-miR-323-3p | 28.9357 | hsa-miR-30d | 27.9315 |
| hsa-miR-197 | 28.9479 | hsa-miR-17 | 27.9378 |
| hsa-miR-20a | 28.9754 | hsa-miR-106a | 27.9389 |
| hsa-miR-193b# | 28.9860 | hsa-miR-92a | 27.9444 |
| hsa-miR-382 | 29.0083 | hsa-miR-663B | 27.9460 |
| hsa-miR-425-5p | 29.0422 | hsa-miR-146b | 27.9516 |
| hsa-miR-151-3p | 29.9330 | hsa-miR-186 | 27.9639 |
| hsa-miR-150 | 29.9471 | hsa-miR-345 | 27.9645 |
| hsa-miR-30e-3p | 29.9707 | hsa-miR-339-3p | 27.9674 |
| hsa-miR-432 | 29.9747 | hsa-miR-193b# | 27.9848 |
|  |  | hsa-miR-202 | 28.0043 |
|  |  | hsa-miR-1225-3P | 28.0675 |
|  |  | hsa-miR-432 | 28.9354 |
|  |  | hsa-miR-629 | 28.9407 |
|  |  | hsa-miR-197 | 28.9556 |
|  |  | RNU48 | 28.9582 |
|  |  | hsa-miR-151-3p | 28.9598 |
|  |  | hsa-miR-323-3p | 28.9635 |
|  |  | hsa-miR-28-3p | 28.9656 |
|  |  | hsa-miR-30a-3p | 28.9758 |
|  |  | hsa-miR-424# | 28.9867 |
|  |  | hsa-miR-874 | 28.9889 |
|  |  | hsa-miR-425-5p | 28.9948 |
|  |  | hsa-miR-382 | 29.0115 |
|  |  | hsa-miR-34b | 29.7833 |
|  |  | hsa-miR-29a | 29.9518 |
|  |  | hsa-miR-375 | 29.9557 |
|  |  | hsa-miR-509-5p | 29.9568 |
|  |  | hsa-miR-203 | 29.9609 |
|  |  | hsa-miR-1227 | 29.9611 |
|  |  | hsa-miR-328 | 29.9665 |
|  |  | hsa-miR-205 | 29.9677 |
